# Mapping cortical disease-burden at individual-level in frontotemporal dementia: implications for clinical care and pharmacological trials

**DOI:** 10.1007/s11682-021-00523-7

**Published:** 2021-12-09

**Authors:** Mary Clare McKenna, Marlene Tahedl, Jasmin Lope, Rangariroyashe H. Chipika, Stacey Li Hi Shing, Mark A. Doherty, Jennifer C. Hengeveld, Alice Vajda, Russell L. McLaughlin, Orla Hardiman, Siobhan Hutchinson, Peter Bede

**Affiliations:** 1 Computational Neuroimaging Group, Biomedical Sciences Institute, Trinity College Dublin, Dublin, Ireland; 2 Department of Psychiatry and Psychotherapy, University of Regensburg, Regensburg, Germany; 3 Institute for Psychology, University of Regensburg, Regensburg, Germany; 4 Complex Trait Genomics Laboratory, Smurfit Institute of Genetics, Trinity College Dublin, Dublin, Ireland; 5 Department of Neurology, St James’s Hospital, Dublin, Ireland

**Keywords:** Frontotemporal dementia, Cerebellum, PPA, Behaviour, MRI, Cortical thickness

## Abstract

Imaging studies of FTD typically present group-level statistics between large cohorts of genetically, molecularly or clinically stratified patients. Group-level statistics are indispensable to appraise unifying radiological traits and describe genotype-associated signatures in academic studies. However, in a clinical setting, the primary objective is the meaningful interpretation of imaging data from individual patients to assist diagnostic classification, inform prognosis, and enable the assessment of progressive changes compared to baseline scans. In an attempt to address the pragmatic demands of clinical imaging, a prospective computational neuroimaging study was undertaken in a cohort of patients across the spectrum of FTD phenotypes. Cortical changes were evaluated in a dual pipeline, using standard cortical thickness analyses and an individualised, z-score based approach to characterise subject-level disease burden. Phenotype-specific patterns of cortical atrophy were readily detected with both methodological approaches. Consistent with their clinical profiles, patients with bvFTD exhibited orbitofrontal, cingulate and dorsolateral prefrontal atrophy. Patients with ALS-FTD displayed precentral gyrus involvement, nfvPPA patients showed widespread cortical degeneration including insular and opercular regions and patients with svPPA exhibited relatively focal anterior temporal lobe atrophy. Cortical atrophy patterns were reliably detected in single individuals, and these maps were consistent with the clinical categorisation. Our preliminary data indicate that standard T1-weighted structural data from single patients may be utilised to generate maps of cortical atrophy. While the computational interpretation of single scans is challenging, it offers unrivalled insights compared to visual inspection. The quantitative evaluation of individual MRI data may aid diagnostic classification, clinical decision making, and assessing longitudinal changes.

## Introduction

The majority of imaging studies in FTD stratifies patients based on clinical, molecular or genetic categories and describes group-specific radiological traits (Omer et al., 2017; Rohrer et al 2011; Whitwell et al., 2005, 2011, 2012). These data however are difficult to apply to individual patients in everyday clinical practice. The current role of MR imaging in the diagnostic pathway of FTD is limited to ‘ruling-out’ structural mimics and alternative diagnoses. MR images acquired in a clinical setting are typically only subjectively and qualitatively interpreted with regards to atrophy (Adachi et al., 2004; Aizpurua et al., 2019; Baez et al., 2014; Campanella et al., 2014; De Maindreville et al., 2015; Di Fede et al., 2019; Harper et al., 2014; Kito et al., 2009; Kotagal et al., 2012; McKeon et al., 2007; Mueller et al., 2018; Muqit et al., 2001; Nishio et al., 2003; Way et al., 2019; Younes et al., 2018). This is a missed opportunity, as raw MRI datasets contain rich, spatially coded information with regards to cortical thickness, subcortical volumes and white matter integrity that cannot be meaningfully appraised on visual inspection. In contrast, computational imaging offers objective, observer-independent, reference-based quantitative image interpretation (Christidi et al., 2018). The potential translation of quantitative MR analysis frameworks to routine clinical practice may offer a number of practical benefits, including the generation of individualised atrophy maps, the objective assessment of longitudinal changes, and the classification of single scans into likely phenotypic categories. Ultimately, quantitative imaging may enable ‘ruling-in’ patients into specific groups, as opposed to merely ‘ruling-out’ differential diagnoses (Bede et al., 2018a, b; Grollemund et al., 2019). From a practical point of view, MR platforms are widely available, MR imaging is non-invasive, relatively cheap, and a multitude of open-source software are available for computational data analyses (Du et al., 2007). Access to 18F-FDG PET imaging on the other hand may be limited and the costs of routine PET imaging may be prohibitive in some health care systems (McMahon et al., 2003; Shivamurthy et al., 2014).

The current diagnostic approach to FTD subtypes—bvFTD, ALS-FTD, nfvPPA, svPPA—requires meeting specific clinical criteria and a definitive diagnosis may only be confirmed in vivo by identifying a pathogenic genetic mutation or typical histopathological findings (Brettschneider et al., 2013; Geser et al., 2009; Gorno-Tempini et al., 2011; Hodges et al., 2010; Perry et al., 2017; Rajagopalan & Pioro, 2015; Rascovsky et al., 2011; Snowden et al., 2007; Strong et al., 2017). The recent development, optimisation and validation of serum and CSF biomarkers panels will not only aid diagnostic classification but help the exclusion of alternative neurodegenerative diagnoses such as Alzheimer’s pathology (Ahmed et al., 2014; Blasco et al., 2018; Devos et al., 2019; Meeter et al., 2019; Paterson et al., 2018; Rascovsky et al., 2011; Steinacker et al., 2017; Swift et al., 2021). As with all diagnostic criteria, there are practical shortcomings with regards to sensitivity and specificity: some symptomatic patients do not meet proposed thresholds for diagnosis, despite subsequent pathological confirmation. In a subset of FTD cases, the diagnosis may never be reached in vivo, or a considerable diagnostic delay is experienced (Harris et al., 2013; Piguet et al., 2009). Diagnostic uncertainty often creates undue stress for the patient and their family. The insidious onset of apathy, lack of interest and social withdrawal may be mistaken for depression, amongst other misdiagnoses (Besser & Galvin, 2020; Rasmussen et al., 2019). Early behavioural symptoms may be difficult to articulate, which is further complicated by the disparity in those perceived by the patients and their caregivers. Early cognitive deficits may also be difficult to identify, particularly due to the masking effect of cognitive reserve and the lack of sensitivity of generic screening instruments (Elamin et al., 2017; Rasmussen et al., 2019). Primary care physicians may reassure patients and caregivers based on neuropsychological screening tests and ‘grossly’ normal MR imaging whilst awaiting lengthy specialist referrals (Rasmussen et al., 2019). Diagnostic delay in neurodegenerative conditions has a number of adverse implications. From a patients’ perspective, timely diagnosis is important to inform realistic expectations over coming years (Spreadbury & Kipps, 2017). It helps to guide targeted genetic testing that may be of significance to other family members. Accurate and early diagnostic classification enables prompt multidisciplinary team referrals and appropriate lifestyle adjustments with regards to employment, finances, driving, and childcare (Spreadbury & Kipps, 2017). In those with language impairment, there is a critical time-window to explore alternative communication options e.g. ‘voice-banking’ to create a digital library for assisted communication devices (Fried-Oken et al., 2015). A timely diagnosis is also important for resource allocation and advanced care planning to ensure that the patients’ end-of-life preferences are recognised (Harrison Dening et al., 2019). Early diagnostic categorisation is also indispensable for the timely inclusion of patients in clinical trials, which in turn enables longer follow-up (Finegan et al., 2019a, b). Based on these considerations, we have undertaken a quantitative imaging study across the spectrum of FTD phenotypes to test a framework to interpret cortical atrophy patterns at both individual- and group-level.

## Methods

### Recruitment

A total of 227 participants were included in this study; 12 patients with non-fluent variant primary progressive aphasia (‘nfvPPA’ 6 females, mean age 61.50 ± 2.97), 3 patients with semantic variant primary progressive aphasia (‘svPPA’ 1 female, mean age 61.67 ± 6.43), 7 patients with behavioural variant FTD (‘bvFTD’ 3 females, mean age 60.71 ± 3.30 years, 20 ALS-FTD patients with *C9orf72* hexanucleotide expansions (‘C9 + ALSFTD’ 8 females, mean age 58.65 ± 11.22), 20 ALS-FTD patients without C9orf72 hexanucleotide expansions (‘C9–ALSFTD’ 7 females, mean age 59.95 ± 7.67), 40 ALS patients with no cognitive impairment (‘ALSnci’ 21 females, mean age 58.70 ± 11.33) as disease controls and 125 healthy controls (HC). Methods for screening for GGGGCC hexanucleotide repeat expansions in *C9orf72* have been previously described (Byrne et al., 2012; Chipika et al., 2020a, b). All participants provided written informed consent in accordance with the ethics approval of the Ethics Medical Research Committee of Beaumont Hospital, Dublin, Ireland. 651 additional HCs were also included from the Cambridge Center for Ageing and Neuroscience (Cam-CAN) data base resulting in a total of 776 healthy controls (HC: 393 females, mean age 55.08 ± 17.63 years) (Shafto et al., 2014).

### Imaging pulse sequences

All local participants were scanned with uniform scanning parameters on a 3 Tesla Philips Achieva scanner using an 8-channel receiver head coil. As described previously (Bede et al., 2019), a 3D Inversion Recovery Prepared Spoiled Gradient Recalled Echo (IP-SPGR) pulse sequence was utilised to acquire T1-weihted images. Acquisition details: repetition time (TR)/echo time (TE) = 8.5/3.9 ms, inversion time (TI) = 1060 ms, field-of-view (FOV): 256 × 256 × 160 mm, spatial resolution: 1 mm^3^. To assess vascular white matter lesion load FLAIR images were also acquired from each participant. The Cam-CAN control subjects were scanned with a T1-weighted MPRAGE sequence on a 3 T Siemens Magnetom TrioTrim scanner at the University of Cambridge, using the following image acquisition parameters: TR/TE 2.25/2.99 ms, TI 900 ms, FOV = 256 × 240 × 192 mm; spatial resolution 1 mm^3^ (Shafto et al., 2014).

### Pre-processing

All subjects’ T1-weighted data were first pre-processed with FreeSurfer’s *recon-all* pipeline to reconstruct and parcellate the cortical surface and generate a cortical thickness (CT) map, which estimates CT at each vertex point of the cortical surface. All CT maps were subsequently transformed to the CIFTI file format at a 32 k resolution per hemisphere (Connectivity Informatics Technology Initiative, (Marcus et al., 2011; Van Essen et al., 2013) using the *Ciftify* toolbox (Dickie et al., 2019). Finally, each subject’s CT map was parcellated into 1000 equally-sized patches, or ‘mosaics’, using a local–global cortical parcellation scheme proposed by (Schaefer et al., 2018), which further refines a previously published 7-brain-network cortical parcellation framework published by (Yeo et al., 2011).

### Statistical analysis: the standard approach

A one-factorial, two-level, between-subjects comparison was first conducted between each patient group and controls controlling for age and gender. To correct for alpha-level inflation, we used a Monte-Carlo permutation procedure to obtain family-wise error-corrected (FWER) p-values (5000 permutations; thresholded at the voxel-level). These analyses were ran within the SPM-based toolbox ﻿(http://www.fil.ion.ucl.ac.uk) *Multivariate and repeated measures* (McFarquhar et al., 2016).

### Statistical analyses: the ‘mosaic’ approach

To appraise cortical thinning at an individual level, each CT map was rated with respect to an age- and sex-matched control group. Since neurite density varies significantly across the cortex (Fukutomi et al., 2018), CT was averaged across small ‘mosaics’, defined by a 1000-patch atlas. For each mosaic, null distributions were built non-parametrically as follows: First, the average CT value of each HC was z-scored with respect to all remaining controls to obtain a distribution at the size of the control group. Likewise, an individual patient’s CT was z-scored with respect to all HC. p-values reflecting expected probabilities of cortical thinning were then calculated by counting how many values in the control distribution were smaller than the observed patient’s and dividing that count by the number of subjects in the control group. We considered mosaics with p-values ≤ 0.05 as significantly thin or ‘atrophic’. To account for confounding effects of age and gender (Trojsi et al., 2020), we customized the reference groups: For each patient, we only included age- and gender-matched controls from the mixed control cohort (in total 776 HC). ‘Age-matched’ was defined as ± 2 years from the patients’ age. As demonstrated before (Tahedl et al., 2021), this strategy successfully corrects for variance introduced by demographic confounders. This strategy generates a binary atrophic/not-atrophic label to each cortical mosaic with reference to demographically matched controls, enables the calculation of the number of ‘significantly thin’ mosaics throughout the cortex, as well as its fraction with respect to all evaluated mosaics. To co-validate the output of this method with the ‘gold standard’ approach we juxtaposed our findings with standard cortical thickness analyses.

### Inferential statistics of ‘mosaic’ maps

The output maps of the mosaic approach can be readily visualized for individual patients indicating whether a cortical region (mosaic) is atrophic (‘hit’) or not with respect of demographically matched controls. (Fig. 2). However, these outputs can also be at group level; we employed a Monte–Carlo permutation testing scheme to compare each of the clinical groups to HCs. In brief, we first generated a matrix with the dimensions of *n*_Patients_ × n_mosaics_ for each clinical group, indicating for each element either the presence (‘1’) or absence (‘0’) of regional atrophy. We then shuffled that matrix 100,000 times across mosaics, whereby we saved the count of patients with 1 s at each iteration. As a result, we obtained non-parametric distributions, comprised of 100,000 values per mosaic, based on which FWER p-values can be calculated by counting the number of values exceeding the observed number of hits in the data and dividing that count by the number of iterations. We considered p-values ≤ 0.05 as statistically significant. Mathematical analyses were conducted within MATLAB version R2019b (The Mathworks, Natick, MA, USA).

### Between group contrasts

Based on the ‘mosaic’ approach, a one-way, six-level analysis of variance (ANOVA) was conducted to ascertain differences among means of whole-brain thin-patch-fractions between the clinical groups. Based on the ‘standard’ approach, the means of raw CT values were also compared with the inclusion of age and gender as covariates (ANCOVA), since, as opposed to the mosaic approach, these are not inherently accounted for. As the ANOVA/ANCOVA revealed statistically significant effects, post-hoc testing was conducted. Tukey’s honestly significant difference testing (HSD) using type III errors were utilised for pairwise comparisons. For post-hoc testing, age was converted into a categorical variable by assigning each patient to one of six separate age groups, since only categorical confounders can be accounted for in Tukey HSD. All statistical analyses were conducted within RStudio (version 1.3.1093, R Core Team, R Foundation for Statistical Computing, Vienna, Austria).

### Region-of-interest statistics

To further characterise regional disease-burden, we calculated fractional thin-patch-counts for four large regions of interest (ROIs): motor cortex (i.e. pre-/paracentral gyri), parietal, temporal and frontal cortices. The 1000-patch mosaic-parcellation was overlaid the anatomically-defined Desikan-Killiany atlas (Fig. 4a) resulting in 122 mosaics in the motor, 185 in the parietal, 150 in the temporal and 200 in the frontal cortices. For each patient, we calculated the fraction of atrophic mosaics, and averaged that fraction across subjects in each clinical subgroup. To highlight the preferential involvement of main brain regions in each phenotype, we generated radar plots (Fig. 4b), in which whole-brain fractional thin-patch-counts were also incorporated. Regional radar plots were also generated to characterise regional involvement in individual patients (Fig. 2).

## Results

**Fig. 1:**
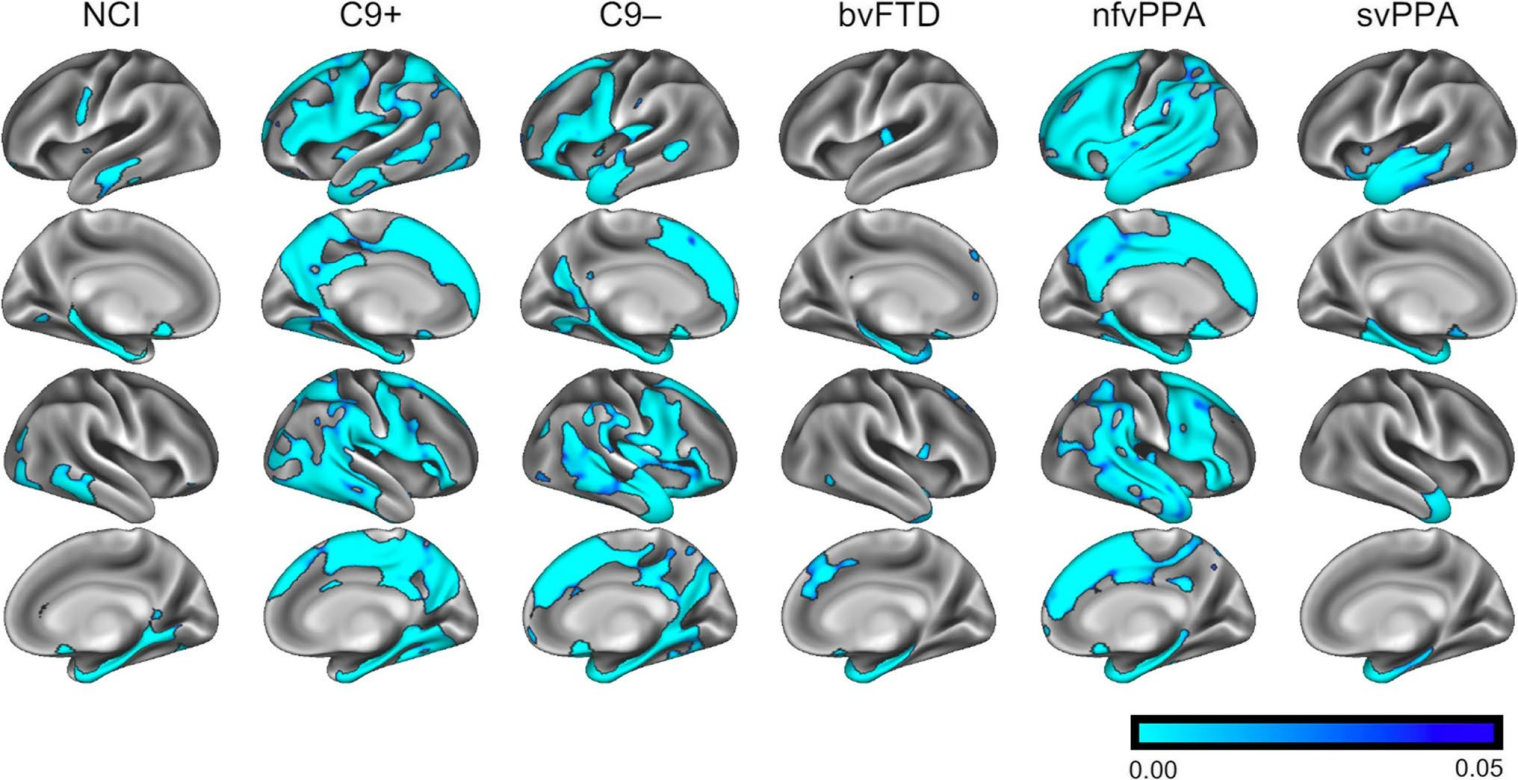
’Standard’ cortical thickness analyses using voxelwise permutation testing, corrected for age and gender; family-wise error corrected p-maps are presented for the six clinical groups with reference to healthy controls. NCI: ALS patients with no cognitive impairment, C9 + : ALS-FTD patients with *C9orf72* hexanucleotide expansions, C9-: ALS-FTD patients without *C9orf72* hexanucleotide expansions, bvFTD: behavioural variant FTD, nfvPPA: non-fluent variant primary progressive aphasia, svPPA: semantic variant primary progressive aphasia

**Fig. 2:**
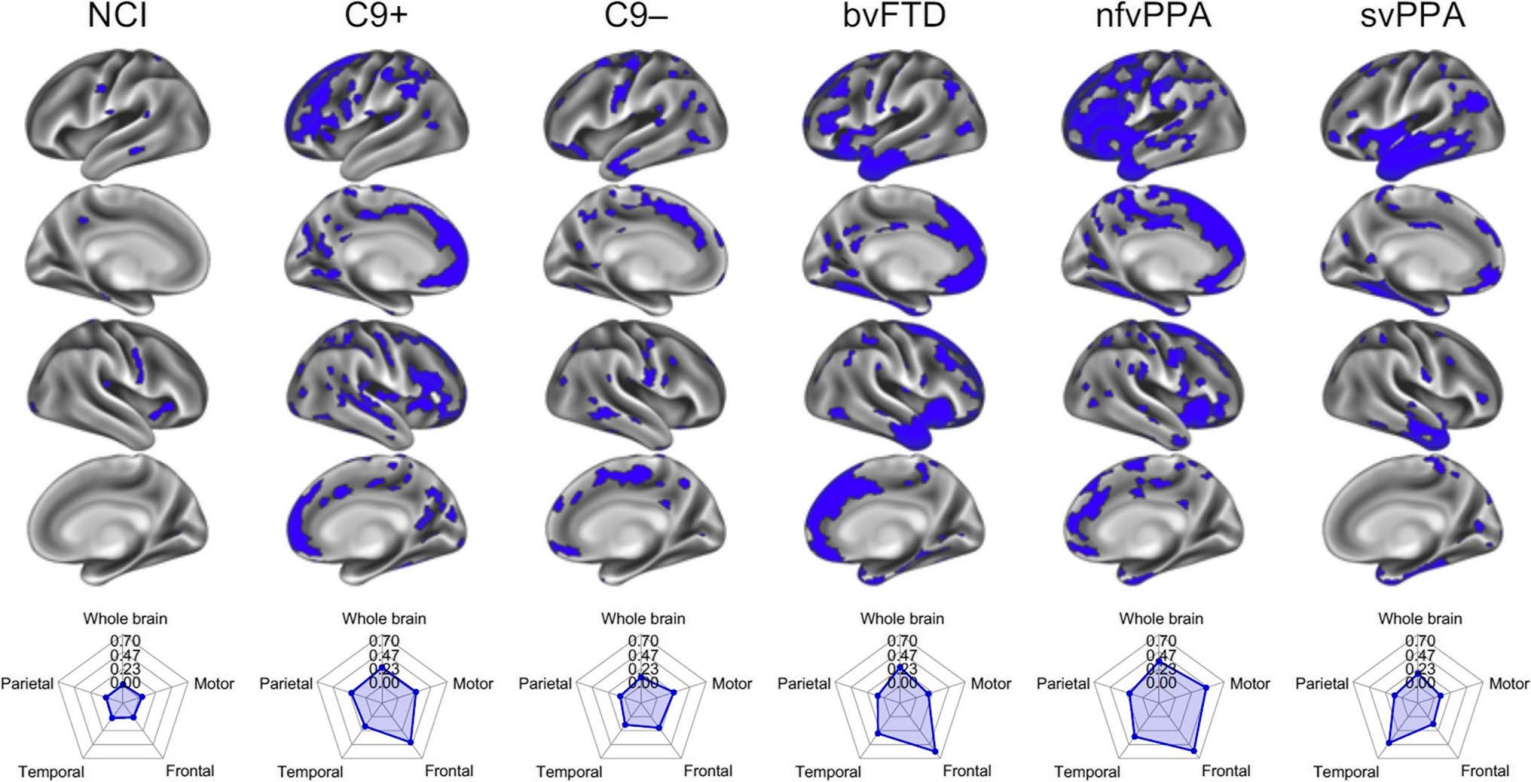
Individual data interpretation in single patients using the ‘mosaic’ pipeline; representative examples are shown from each clinical groups. Blue colour indicates cortical thinning with respect to demographically matched controls. Radar charts indicate the fraction of affected ‘mosaics’ in frontal, parietal, temporal and motor cortices as well as over the entire cortex. NCI: ALS patients with no cognitive impairment, C9 + : ALS-FTD patients with *C9orf72* hexanucleotide expansions, C9-: ALS-FTD patients without *C9orf72* hexanucleotide expansions, bvFTD: behavioural variant FTD, nfvPPA: non-fluent variant primary progressive aphasia, svPPA: semantic variant primary progressive aphasia

**Fig. 3:**
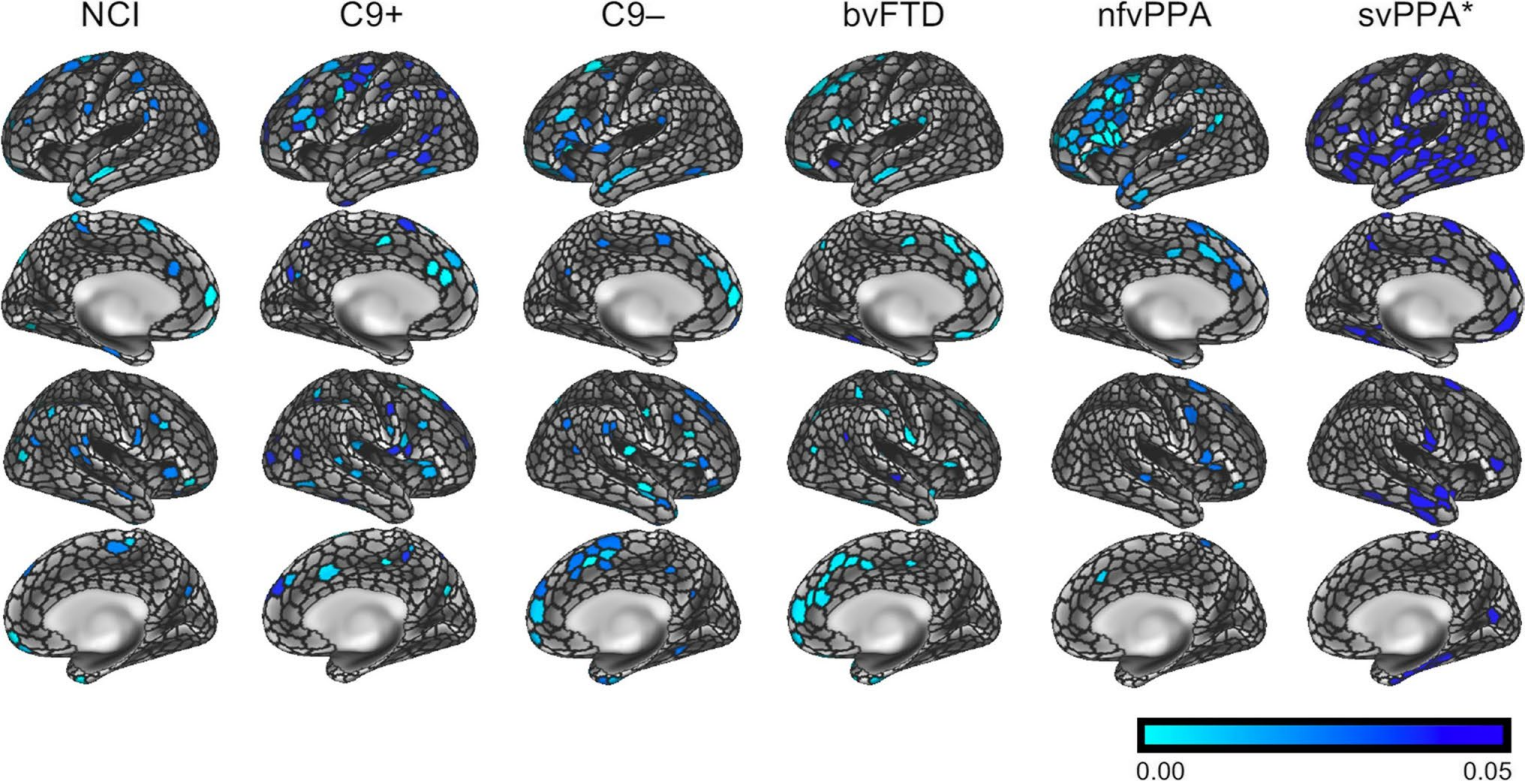
Inferential statistics; group-level atrophy patterns derived from the ‘mosaic’ approach. Family-wise error-corrected p-maps are presented at p < 0.05. For svPPA a threshold of p < 0.06 is shown. NCI: ALS patients with no cognitive impairment, C9 + : ALS-FTD patients with *C9orf72* hexanucleotide expansions, C9-: ALS-FTD patients without *C9orf72* hexanucleotide expansions, bvFTD: behavioural variant FTD, nfvPPA: non-fluent variant primary progressive aphasia, svPPA: semantic variant primary progressive aphasia

Standard cortical thickness analyses confirmed subgroup-specific patterns of cortical atrophy consistent with the clinical diagnosis (Fig. 1). The ‘mosaic-based’ approach has successfully generated individual atrophy maps for each patient with reference to controls (Fig. 2). Group-level observations could also be inferred from the ‘mosaic-based’ approach following permutation testing (Fig. 3). These results were anatomically consistent with the outputs of the ‘standard approach’ (Fig. 1). Group-level traits deduced from the ‘mosaic-based’ approach produced more focal and better demarcated atrophy maps than those generated by the standard approach. This is best demonstrated by the C9 + ALS-FTD group where atrophy is not just more widespread than the C9–ALS-FTD group, but the precentral gyrus is more affected. Cortical atrophy patterns derived from the ‘mosaic-approach’ are also more focal and less noisy in the nfvPPA group than the in the maps generated by the standard approach.

**Fig. 4:**
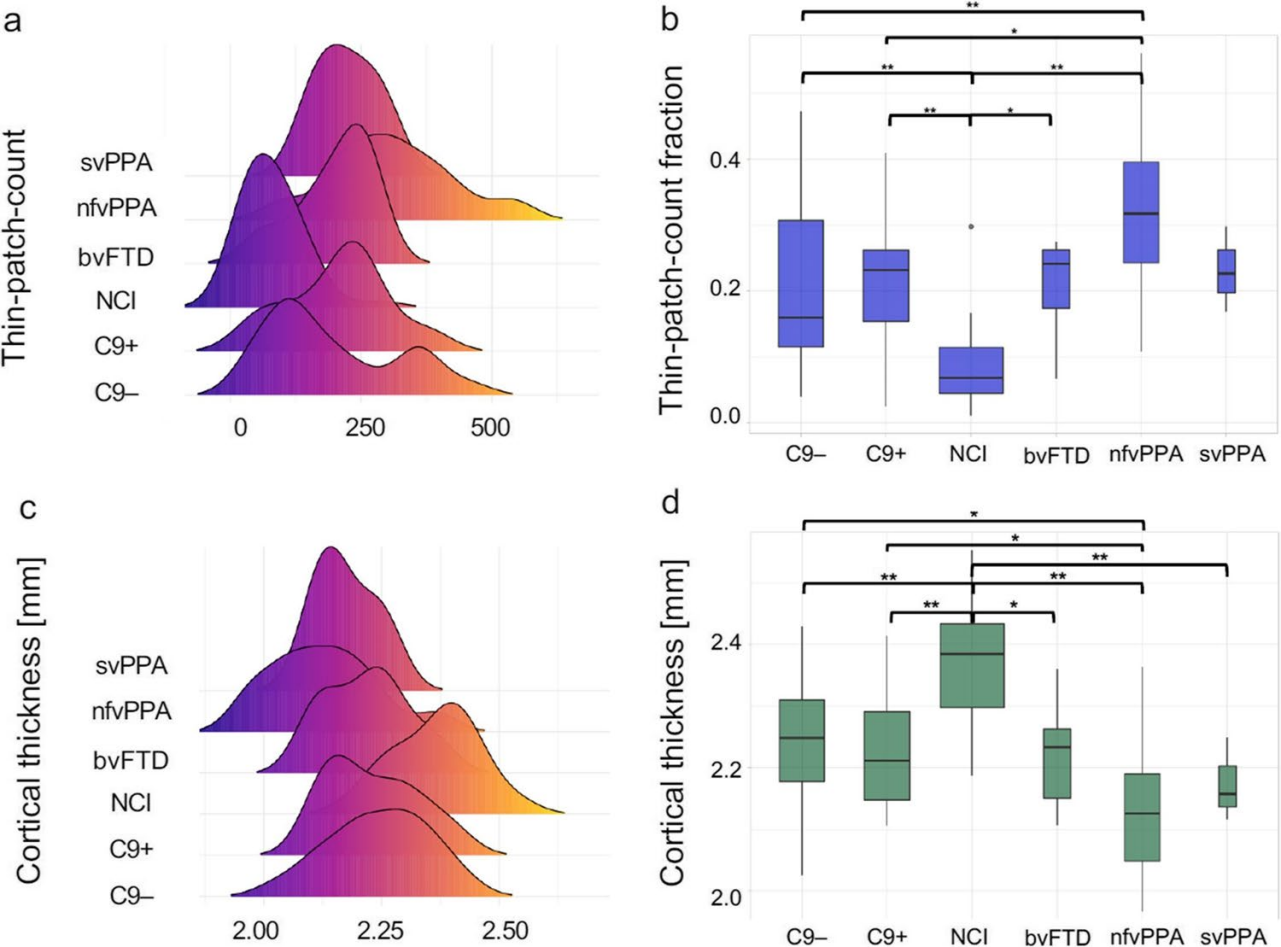
The comparison of group profiles; distribution of the number of thin patches derived from the ‘mosaic approach’ (**a**) and cortical thickness values as calculated by the ‘standard approach’ (**c**). Group differences in the number of thin patches (**b**) and mean cortical thickness (**d**). * indicates post hoc intergroup difference at p_*adj*_ ≤ 0.05, (**) at p_*adj*_ ≤ 0.001 following Tukey HSD testing. The widths of box plots indicate sample size and error bars represent 1.5 times the interquartile range. NCI: ALS patients with no cognitive impairment, C9 + : ALS-FTD patients with C9orf72 hexanucleotide expansions, C9-: ALS-FTD patients without C9orf72 hexanucleotide expansions, bvFTD: behavioural variant FTD, nfvPPA: non-fluent variant primary progressive aphasia, svPPA: semantic variant primary progressive aphasia

Both the ‘mosaic’ and the ‘standard’ approach indicated intergroup differences (Fig. 4a, c) (mosaic approach: *F*(5) = 14.86, *p* = 8.73e−11; standard approach: *F*(5) = 14.89, *p* = 9.50e−11). Post-hoc testing revealed that least affected study group was ALSnci compared to all the other diagnostic categories. (Fig. 4b) ALSnci vs. C9–(0.202 ± 0.132), *p*_*adj*_ = 1.76e−04; ALSnci versus C9 + (0.214 ± 0.100), *p*_*adj*_ = 2.54e−05; ALSnci versus bvFTD (0.208 ± 0.076), *p*_*adj*_ = 2.01e−02; ALSnci vs. nfvPPA (0.321 ± 0.121), *p*_*adj*_ < 0.0001. The same pattern was observed for the standard approach (Fig. 4d), where the ALSnci group exhibited higher CT in the pairwise comparisons than all other groups: ALSnci versus C9–(2.24 mm ± 0.11 mm), *p*_*adj*_ = 1.85e−04; ALSnci versus C9 + (2.23 mm ± 0.10 mm), *p*_*adj*_ = 2.86e−05; ALSnci versus bvFTD (2.22 mm ± 0.09 mm), *p*_*adj*_ = 4.30e−03; ALSnci versus nfvPPA (2.13 mm ± 0.11 mm), *p*_*adj*_ < 0.0001; ALSnci versus svPPA (2.17 mm ± 0.07 mm), *p*_*adj*_ = 1.61e−02. In contrast, the most affected clinical group was nfvPPA, where the mean thin-patch-count fraction was not only higher than that of the ALSnci group, but also the C9–ALSFTD (*p*_*adj*_ = 9.45e−03) and the C9 + ALSFTD (*p*_*adj*_ = 2.80e−02). Again, this pattern was mirrored by the standard approach, where the ALSnci group not only showed higher mean values than the nfvPPA group, but just as in the mosaic approach, also the C9–ALSFTD (*p*_*adj*_ = 1.72e−02) and the C9 + ALSFTD (*p*_*adj*_ = 4.66e−02) groups.

**Fig. 5:**
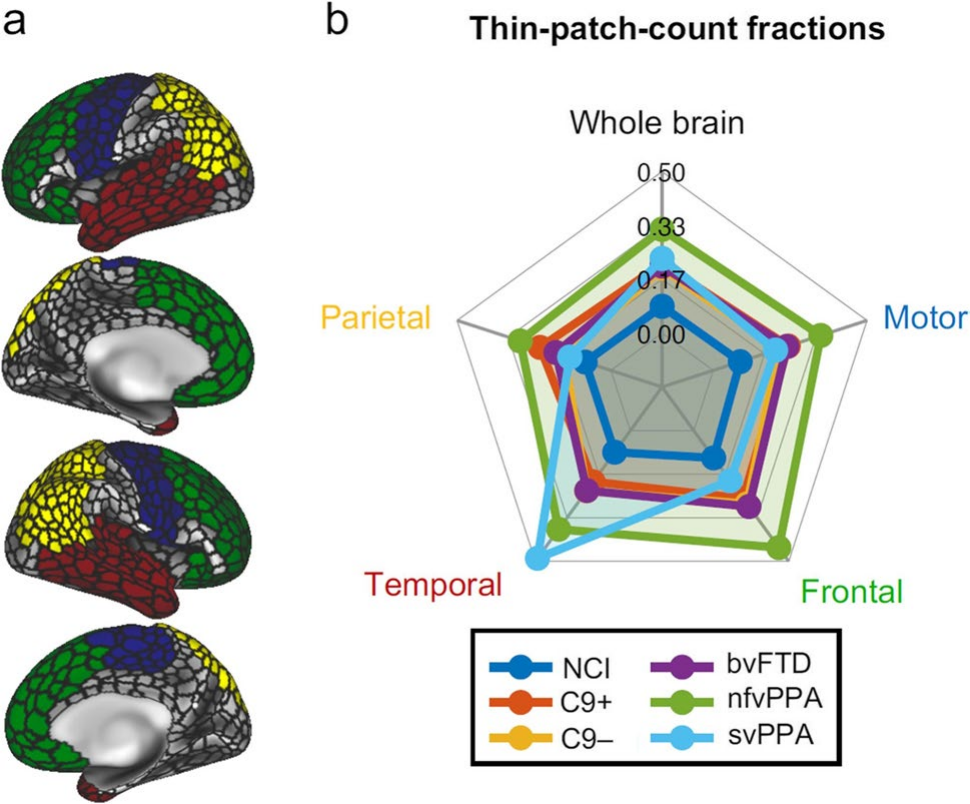
Regional disease burden; cortical thinning was further evaluated in four atlas-defined regions-of-interest (ROIs) in the motor (blue), parietal (yellow), temporal (red) and frontal (green) cortices and over the entire cerebral cortex (**a**). The fraction of atrophic ‘mosaics’ was calculated in each patient within each ROI with respect to the total number of mosaics comprising the given ROI. The distribution of disease burden in the patient groups is presented as radar charts (**b**). NCI: ALS patients with no cognitive impairment, C9 + : ALS-FTD patients with C9orf72 hexanucleotide expansions, C9-: ALS-FTD patients without C9orf72 hexanucleotide expansions, bvFTD: behavioural variant FTD, nfvPPA: non-fluent variant primary progressive aphasia, svPPA: semantic variant primary progressive aphasia

Our region-of-interest statistics evaluated thin-patch-count fraction per ‘ROI’ (Fig. 5a) and confirmed the preferential involvement of ROIs in the study groups (Fig. 5b). The most anatomically widespread disease-burden was detected in nfvPPA (largest radius), the least pathology in ALSnci (smallest radius) and the most focal involvement in svPPA (temporal cortex).

## Discussion

Our findings demonstrate the feasibility of interpreting single T1-weighted images from single patients and generating individual maps of atrophy. We have shown that cortical regions can be successfully categorised as atrophic or unaffected in single subjects with respect to a databank of controls. A z-score based approach not only enables the appraisal of cortical disease-burden in individual-subjects, but group-level patterns may also be inferred. The output maps of the proposed ‘mosaic’ approach are anatomically concordant with gold standard cortical thickness analyses. The topography of cortical thinning can be reported visually, numerically and in an ROI-based representation at both individual- and group-level. The pipeline is based on quantitative cortical thickness measurements, an atlas-based parcellation and is fully observer independent. In its current form it is computationally demanding, but all the mathematical steps utilised could be integrated into a single computer script and run either as a cloud-based solution or installed locally on the MR platform or data server.

In this paper we have demonstrated the utility of this approach in FTD phenotypes, but this method could potentially also be utilised in neurodegenerative conditions where the ascertainment of cortical atrophy patterns is clinically relevant (Abidi et al., 2020a, b; Christidi et al., 2019; Finegan et al., 2019a, b; Nasseroleslami et al., 2019; Seo et al., 2010). The technique relies on the binary labelling of cortical regions as ‘atrophic’ or ‘normal’. This is fundamentally a reductionist approach, but given the very high number of cortical regions ('mosaics'), it is a successful strategy as demonstrated by the detection of confluent cortical areas. The generation of putative atrophy maps provides an instant representation of the anatomical expansion, focality and lobar predominance of disease burden. These colour coded maps are potentially useful to illustrate affected regions to patients, caregivers and members of the multidisciplinary team. This starkly contrasts with the current practice of pointing at presumed regions of atrophy on black and white 2D images which are difficult to decipher by laypeople (Harper et al., 2014). The z-score derived, ‘mosaic’ method may not only be applied to those with an established diagnosis, but also to those with a suspected diagnosis or pre-symptomatic mutation carriers to characterise disease burden distribution.

In a clinical setting, progressive frontotemporal pathology is often monitored by validated neuropsychological tests (Burke et al., 2016a, b, 2017; Elamin et al., 2017). Cognitive assessment however may be particularly challenging in certain FTD phenotypes, especially in ALS-FTD where motor disability and dysarthria may preclude the use of certain tests (Burke et al., 2016a, b; Verstraete et al., 2015; Yunusova et al., 2019). In other FTD phenotypes, performance on neuropsychological testing may be confounded by mood, apathy, cognitive reserve and practice-effects which support the role of neuroimaging in tracking progressive changes objectively (Costello et al., 2021; Radakovic et al., 2016).

Quantitative cortical thickness mapping may also give additional reassurance to those who fear a particular diagnosis despite scoring high on neuropsychological tests (Hardiman et al., 2016). This is often a significant source of anxiety for patients, particularly for those who have first-hand witnessed a family member or close friend carrying a certain a diagnosis. Immediate answers would provide early reassurance, alleviating the sense of heightened stress and anxiety. The implementation of this method may be relatively straightforward as most patients undergo a routine MRI brain scan as part of the diagnostic pathway (Harper et al., 2014).

Despite the clinical rationale to devise such frameworks, our study has a number of limitations. The sample size of the various patient groups is relatively small in this study necessitating validation in larger external datasets. All patients in our study had an established diagnosis; thus, the sensitivity of this method needs to be further evaluated in those with a suspected diagnosis, early-stage disease or in asymptomatic mutation carriers (Chipika et al., 2020a, 2020b; Li Hi Shing et al., 2021; Querin et al., 2019a, b). Moreover, only grey matter analyses were conducted, despite the contribution of white matter pathology to the clinical manifestations of these phenotypes (Bede et al., 2016, 2018a, b; Qin et al., 2021; Schuster et al., 2016a, b; Zhou et al., 2010). Finally, while our approach provides individualised atrophy maps, supervised and unsupervised machine learning approaches offer direct individual patient categorisation into diagnostic and prognostic groups (Bede et al., 2017; Grollemund et al., 2020a, b; Grollemund et al., 2020a, b; Querin et al., 2018; Schuster et al., 2016a, b).

We envisage future applications for this methodological approach in both clinical practice and potentially in clinical trials. Consecutive MR datasets could be compared to the patients’ initial scan; allowing for the objective measurement of disease-burden accumulation and the evaluation of progression rates (Bejanin et al., 2020; Chipika et al., 2019; Schuster et al., 2015). Alternative imaging metrics such as spinal cord measures, network integrity indices, white matter diffusivity parameters or subcortical grey matter metrics could also be readily investigated in a similar z-score based framework (Abidi et al., 2020a, b; Dukic et al., 2019; El Mendili et al., 2019; Proudfoot et al., 2018; Querin et al., 2019a, b). Future applications would require the validation of our findings in large multicentre studies, ideally incorporating diverse patient populations across a variety of neurodegenerative disorders.

## Conclusions

Our preliminary findings indicate that T1-weighted MRI data from individual patients may be meaningfully interpreted and maps of cortical atrophy can be readily generated for single patients. The outputs of our pipeline are anatomically analogous with gold standard methods. The presented framework offers a viable quantitative approach to interpret single subject scans with practical clinical utility and potential for clinical trial applications.

## Data Availability

Raw imaging data cannot be shared due to institutional policies. Additional information on processing pipelines may be requested from the corresponding author.
